# Association of dietary flavan-3-ol intakes with plasma phenyl-γ-valerolactones: analysis from the TUDA cohort of healthy older adults

**DOI:** 10.1016/j.ajcnut.2023.06.006

**Published:** 2023-06-10

**Authors:** Donato Angelino, Aoife Caffrey, Helene McNulty, Chris IR. Gill, Pedro Mena, Alice Rosi, Katie Moore, Leane Hoey, Michelle Clements, Eamon Laird, Kerrie Boyd, Brian Mullen, Bruna Pucci, Harry Jarrett, Conal Cunningham, Mary Ward, J.J. Strain, Kevin McCarroll, Adrian J. Moore, Anne M. Molloy, Daniele Del Rio

**Affiliations:** 1 Human Nutrition Unit, Department of Food and Drug, University of Parma, Parma, Italy; 2 Department of Bioscience and Technology for Food, Agriculture, and Environment, University of Teramo, Teramo, Italy; 3 Nutrition Innovation Centre for Food and Health (NICHE), School of Biomedical Sciences, Ulster University, Coleraine, Northern Ireland, UK; 4 Microbiome Research Hub, University of Parma, Parma, Italy; 5 Department of Clinical Medicine, School of Medicine, Trinity Centre for Health Sciences, Trinity College Dublin, Ireland; 6 School of Geography and Environmental Sciences, Ulster University, Coleraine, Northern Ireland, UK

**Keywords:** Dietary (poly)phenols, flavan-3-ols, procyanidins, (epi)catechins, phenyl-γ-valerolactones, older adults

## Abstract

**Background:**

Dietary polyphenols, including flavan-3-ols (F3O), are associated with better health outcomes. The relationship of plasma phenyl-γ-valerolactones (PVLs), the products of colonic bacterial metabolism of F3O, with dietary intakes is unclear.

**Objectives:**

To investigate whether plasma PVLs are associated with self-reported intakes of total F3O and procyanidins+(epi)catechins.

**Design:**

We measured 9 PVLs by uHPLC-MS-MS in plasma from adults (>60y) in the Trinity-Ulster-Department of Agriculture (TUDA study (2008 to 2012; n=5186) and a follow-up subset (2014 to 2018) with corresponding dietary data (n=557). Dietary (poly)phenols collected by FFQ were analyzed using Phenol-Explorer.

**Results:**

Mean (95% confidence interval [CI]) intakes were estimated as 2283 (2213, 2352) mg/d for total (poly)phenols, 674 (648, 701) for total F3O, and 152 (146, 158) for procyanidins+(epi)catechins. Two PVL metabolites were detected in plasma from the majority of participants, 5-(hydroxyphenyl)-γ-VL-sulfate (PVL1) and 5-(4ʹ-hydroxyphenyl)-γ-VL-3ʹ-glucuronide (PVL2). The 7 other PVLs were detectable only in 1-32% of samples. Self-reported intakes (mg/d) of F3O (*r* = 0.113, *P* = 0.017) and procyanidin+(epi)catechin (*r* = 0.122, *P* = 0.010) showed statistically significant correlations with the sum of PVL1 and PVL 2 (PVL1+2). With increasing intake quartiles (Q1-Q4), mean (95% CI) PVL1+2 increased; from 28.3 (20.8, 35.9) nmol/L in Q1 to 45.2 (37.2, 53.2) nmol/L in Q4; *P* = 0.025, for dietary F3O, and from 27.4 (19.1, 35.8) nmol/L in Q1 to 46.5 (38.2, 54.9) nmol/L in Q4; *P* = 0.020, for procyanidins+(epi)catechins.

**Conclusions:**

Of 9 PVL metabolites investigated, 2 were detected in most samples and were weakly associated with intakes of total F3O and procyanidins+(epi)catechins. Future controlled feeding studies are required to validate plasma PVLs as biomarkers of these dietary polyphenols.

## Introduction

Epidemiological studies show that higher consumption of fruit and vegetables is associated with better health outcomes [1]. Apart from providing a source of various micronutrients, these plant-based foods are rich in bioactive compounds, including (poly)phenols, the intake of which is associated with a reduced risk of cardiovascular and neurodegenerative diseases [2]. Among the flavonoids, a large family of (poly)phenols, flavan-3-ols (F3O), are of particular interest, with the findings of intervention studies suggesting beneficial roles in improving cognitive function [3,4] and preventing cardiovascular disease events, as recently reported in the COSMOS trial [5].

Although F3O-rich foods and other dietary (poly)phenols are widely associated with better cognitive health, the concentrations at which such bioactive compounds may act as biological effectors in the brain are still to be established. Furthermore, bioavailability studies have confirmed that only a small fraction of ingested F3O is absorbed in the upper gastrointestinal tract, mainly as monomers and structurally related (epi)catechin metabolites (SREM), that reach peak concentrations in the bloodstream at around 2 h and are excreted within 8 h after ingestion [6,7]. The remaining F3O fraction reaches the colonic tract and undergoes an extensive gut microbial metabolism, leading to smaller compounds, including phenolic acids and phenyl-γ-valerolactones (PVLs) [8]. These smaller metabolites, mainly in their sulfated, glucuronidated, and methylated forms, have been shown to reach the bloodstream in micromolar concentrations, with a T_max_ of ∼6 h, and remain in circulation well beyond 24 h [7,9]. Our previous studies demonstrated that PVL conjugates can cross the blood-brain barrier and enter brain cells [10], inducing inhibition of the formation of β-amyloid aggregates in vitro, with consequent reduction of their cerebral toxic effects *in vivo* [11]. The different pharmacokinetic and excretion profiles of SREMs and PVLs raise the issue as to which forms might best reflect exposure to F3O-rich food to supplement dietary intake assessments for research in this area [12]. Ottaviani et al. demonstrated that both classes of compounds reflect exposure to dietary F3O intake, providing complementary information on monomer and oligomer/polymer intakes [13,14]. However, compared with SREMs, the longer persistence in the circulation of PVLs makes them potentially more attractive for studies of long-term F3O intake [14]. Also, given the biological activities attributed to PVLs, measurement of these metabolites could provide a basis to study the mechanistic link between F3O exposure and cognitive performance.

The overall purpose of the transnational Valerolactones and Healthy Ageing (VALID) project was to examine the relationship of dietary polyphenols with health outcomes in older age [15]. There is, however, little information available in the literature regarding the association of dietary polyphenols with PVL metabolites. For this reason, as a part of the wider VALID project, the current study aimed to investigate whether plasma PVLs were associated with self-reported measures of dietary F3O and procyanidins+(epi)catechins intakes in a large cohort of free-living older adults.

## Methods

### Participants

This observational study involved new analysis of blood samples and the assessment of habitual (poly)phenol intakes in participants from the Trinity-Ulster-Department of Agriculture (TUDA) study. This cohort (n = 5186) was first sampled from 2008 to 2012, and a proportion (20%) was reinvestigated from 2014 to 2018. Procedures for the recruitment and sampling of the original TUDA cohort of adults aged ≥60 y are described in detail elsewhere [16]. Briefly, TUDA participants were initially recruited from hospital outpatient or General Practice clinics from 2 jurisdictions, Northern Ireland (UK) and the Republic of Ireland, and were deemed eligible for inclusion if they were without a diagnosis of dementia and either they or their parents were born on the island of Ireland. Ethical approval for the original study and all follow-up investigations was obtained from the Office for Research Ethics Committees Northern Ireland (ORECNI; ref. 08/NIR03/113), with corresponding approvals from the Northern and Western Health and Social Care Trusts in Northern Ireland, and the Research Ethics Committee of St James’s Hospital and The Adelaide and Meath Hospital, Dublin, Republic of Ireland. All TUDA participants provided informed consent at the time of initial recruitment and for follow-up investigations, and the study was registered at Clinicaltrials.gov (identifier NCT02664584).

For the current investigation, TUDA participants from the original study and those participants who were reinvestigated from 2014 to 2018 were considered. The exclusion criteria for the follow-up investigation were age < 65 y, a Mini-Mental State Examination score < 21 at initial sampling, or receiving vitamin B12 injections. From the available TUDA sample meeting the criteria for follow-up investigation, 953 were willing to participate across centers in Northern Ireland and the Republic of Ireland. Of these, participants who completed a food frequency questionnaire (FFQ) for dietary polyphenols and provided a blood sample were eligible for inclusion in the current analysis (Supplementary Figure 1).

### Dietary assessment of the TUDA cohort

In the TUDA study follow-up investigation (but not at baseline), dietary intake data were collected using a 4-day food diary (which included 2 weekdays and 2 weekend days) in combination with a FFQ assessing the past year. This method is described in more detail elsewhere [17]. Dietary records were collected by trained nutritionists using standardized protocols, and one dietitian oversaw training for dietary data quality assurance. Each participant received oral and written instructions on how to complete the food diary and FFQ. On collection of the food diary and FFQ, records were reviewed by the team, and any queries or discrepancies between the 2 dietary records were discussed and clarified with the participant. Food portion sizes were estimated by the participant using household measures and quantified using published food portion size data available in Nutritics (Version 5.76; Research Edition, Dublin, Ireland; www.nutritics.com). Mean daily energy and macronutrient intakes were calculated using the Nutritics nutrition analysis software. Potential misreporting was identified via an assessment of reported energy intake (EI) and estimated energy requirements (EER) based on the combination of Basal Metabolic Rate (BMR) and Physical Activity Level (PAL), using the following calculation: (EI – EER) / EER x 100, a formula which makes the assumption that weight is stable. BMR and PAL were estimated using the sex-specific Oxford equations for BMR [18] and predicted values of PAL [19]. Potential misreporting (over- or under-reporting) of dietary EI was identified using the threshold value of +/- 30% of the EER value for each individual, as previously described [20].

Specifically for the assessment of habitual (poly)phenol intakes, an FFQ only was used and designed as an interviewer-led semi-quantitative FFQ (Supplementary Figure 2). The development of the FFQ was informed by Cade et al. [21], and the design was modeled on the FFQ from the European Prospective Investigation into Cancer and Nutrition (EPIC) study in terms of frequency categories and portion sizes [22]. Further adaptations included a shortened frequency scale (‘rarely to never,’ ‘a few times a month,’ ‘once a week or more,’ and ‘daily’), with open-ended questions for foods/beverages commonly consumed more than once a day (e.g., tea and coffee). Open-ended sections were also included to record locally consumed foods that may contain (poly)phenols and are not necessarily accounted for in the FFQ.

A database of (poly) phenol-containing foods was generated using Phenol-Explorer, the first comprehensive database on polyphenol content in foods that contains more than 35,000 content values for 500 different (poly)phenols in over 400 foods [(23]; accessed May 2019). This database was also supplemented with values from the USDA databases of flavonoid and proanthocyanidin content of specific foods [24] that were not included in Phenol-Explorer. Where available, recipes were calculated for some common basic foods (e.g., breakfast muesli), and retention and yield factors were applied to account for changes in (poly)phenol content from cooking (e.g., values for cooked versus raw kidney beans). Thereafter, foods were ranked by serving size obtained from the Food Standards Agency [25], food packaging, or published sources [26], and included those foods providing >1 mg of F3O per serving that were identified as being relevant to TUDA study participants. Additional foods were included if they contributed relatively low quantities of (poly)phenols yet were consumed with relatively high frequency within the TUDA cohort (e.g., coffee). Where multiple varieties of the same food were present, the variety most applicable to the demographic was included (e.g., black tea infusion instead of bottled black tea), or the most simplified description was used (e.g., a generic apple instead of specific varieties such as Granny Smith, Golden Delicious, etc.). Neither herbs or spices nor differences between peeled or unpeeled fruits were considered. Finally, open-ended responses were calculated and included as recipes (e.g., mixed nuts) or estimations that were based on similar food items where calculations were not possible (e.g., fresh blueberries ≥ dried). Dietary intakes were estimated for total dietary (poly)phenols, major subgroups as i) total F3O, ii) total theaflavins and thearubigins, iii) total proanthocyanidins and the relative monomers, as (epi)catechins and their gallate esters, (epi)gallocatechins and their gallate esters, iv) procyanidins, and their sub-components as catechin and epicatechin.

### Lifestyle and anthropometric measures

In the original and follow-up TUDA studies, a comprehensive health and lifestyle questionnaire was administered as part of a 90-min interview to capture general health, medical and demographic details and included information on medications, smoking status, alcohol consumption, and vitamin supplement usage. Height and weight for each participant were measured by using portable approved scales (Seca; Brosch Direct Ltd, Peterborough, United Kingdom), and body mass index was calculated. Waist and hip measurements were recorded in accordance with standard operating procedures.

### Laboratory analysis

#### Plasma phenyl-γ-valerolactone analysis

For all blood samples, processing was carried out according to standard protocols within 4 h of collection, and plasma samples were stored at −70 °C until batch analysis.

Phenyl-γ-valerolactone metabolites were extracted and concentrated using a solid phase extraction method, as previously reported [27], and were analyzed by a UHPLC DIONEX Ultimate 3000 equipped with a triple quadrupole TSQ mass spectrometer fitted with a heated-ESI (H-ESI) probe (Thermo Fisher Scientific Inc., San José, CA, USA) at the University of Parma, Italy. Chromatographic separation, ionization parameters, and spectrometric characteristics of the considered compounds are shown in Table 1 and in Supplementary Method. A total of 9 PVLs were measured in plasma using UHPLC-ESI-MS/MS: 5-(hydroxyphenyl)-γ-valerolactone-sulfate, which represents the sum of 2 isomers coeluting, 5-(3′-hydroxyphenyl)-γ-valerolactone-4′-sulfate and 5-(4′-hydroxyphenyl)-γ-valerolactone-3′-sulfate, PVL1; 5-(4ʹ-hydroxyphenyl)-γ-valerolactone-3ʹ-glucuronide, PVL2; 5-(5ʹ-hydroxyphenyl)-γ-valerolactone-3ʹ-glucuronide, PVL3; 5-phenyl-γ-valerolactone-4ʹ-sulfate, PVL4; 5-phenyl-γ-valerolactone-3ʹ-sulfate, PVL5; 5-phenyl-γ-valerolactone-3ʹ-glucuronide, PVL6; 5-(5ʹ-hydroxyphenyl)-γ-valerolactone-3ʹ-sulfate, PVL7; 5-(3ʹ,4ʹ-dihydroxyphenyl)-γ-valerolactone, PVL8; 5-(3ʹ,5ʹ-dihydroxyphenyl)-γ-valerolactone, PVL9. However, PVL8 and PVL9 were not detected in this cohort and thus were not included in any further analysis. In the absence of appropriate internal standards, quantification was performed with calibration curves of standards, when available, or using the most structurally similar compound [28,29]. Samples were analyzed in different batches and times, analytical quality was monitored by rerunning pooled quality control samples from different batches throughout the analysis. The inter-day precision value, as respective relative standard deviation (% RSD), was set at 10% for rejection of the results. Data processing was performed using Xcalibur software (Thermo Scientific Inc., Waltham, MA, USA). For each PVL, concentrations less than the lower limit of quantitation (LLOQ) were reported as LLOQ/2 [30,31].

**TABLE 1:** Phenyl-γ-valerolactone metabolites for identification and quantification in human plasma samples by UHPLC-ESI-MS/MS^1^

Phenyl-γ-valerolactone metabolites		RT (min)	Parent ion (*m/z*)	S-lens	Quantifier		Qualifier		LOD (nmol/L)	LLOQ (nmol/L)
					Product ion (*m/z*)	CE (V)	Product ion (*m/z*)	CE (V)		
**PVL1**	5-(Hydroxyphenyl)-γ-valerolactone-sulfate^2^	3.90	287	96	207	24	163	23	0.8	2.5
**PVL2**	5-(4ʹ-Hydroxyphenyl)-γ-valerolactone-3ʹ-glucuronide^3^	3.90	383	93	207	23	163	27	3.0	7.5
**PVL3**	5-(5ʹ-Hydroxyphenyl)-γ-valerolactone-3ʹ-glucuronide	3.70	383	93	163	23	163	27	3.0	7.5
**PVL4**	5-phenyl-γ-valerolactone-4ʹ-sulfate	4.78	271	93	191	23	147	21	2.0	6.0
**PVL5**	5-phenyl-γ-valerolactone-3ʹ-sulfate	4.95	271	93	191	25	106	21	2.0	6.0
**PVL6**	5-phenyl-γ-valerolactone-3ʹ-glucuronide	4.20	367	93	191	25	147	20	15.0	30.0
**PVL7**	5-(5ʹ-Hydroxyphenyl)-γ-valerolactone-3ʹ-sulfate	2.13	287	96	207	23	163	23	6.3	12.3
**PVL8**	5-(3ʹ,4ʹ-Dihydroxyphenyl)-γ-valerolactone^4^	4.04	207	75	163	20	122	30	6.2	12.4
**PVL9**	5-(3ʹ,5ʹ-Dihydroxyphenyl)-γ-valerolactone^4^	3.64	207	75	123	21	163	30	61	123

1 Chromatographic separation, ionization parameters, and spectrometric characteristics of the considered compounds. CE, collision energy; LLOQ, lower limit of quantification; LOD, limit of detection; RT, retention time; UHPLC-ESI-MS/MS, ultrahigh performance liquid chromatography–electrospray ionization tandem mass spectrometry.

2 5-(Hydroxyphenyl)-γ-valerolactone-sulfate (PVL1) is the sum of two isomers coeluting, 5-(3′-hydroxyphenyl)-γ-valerolactone-4′-sulfate and 5-(4′-hydroxyphenyl)-γ-valerolactone-3′-sulfate.

3 The compound 5-(4ʹ-hydroxyphenyl)-γ-valerolactone-3ʹ-glucuronide (PVL2) has been quantified as 5-(5ʹ-hydroxyphenyl)-γ-valerolactone-3ʹ-glucuronide (PVL3).

4 These metabolites were not detected in this cohort.

### Statistical analysis

All statistical analyses were carried out using SPSS software (version 26; SPSS UK Ltd, Chertsey, United Kingdom). For the current investigation, TUDA participants from the original study and those who were reinvestigated from 2014 to 2018 were considered. Differences in general characteristics between males and females were assessed using independent samples t test (continuous variables) or chi-square test (categorical variables). Differences in dietary and PVL data were analyzed by analysis of covariance (ANCOVA), adjusting for age, on log-transformed data where appropriate. For the analysis of dietary-plasma PVL associations, the data were examined using 2 approaches. First, dietary polyphenol and PVL data were analyzed by bivariate correlations using Spearman correlation coefficient and partial correlations with adjustment for age, sex, and dietary energy (kcals/d). Second, data were analyzed by using ANCOVA with the Least Significant Difference post hoc test after splitting dietary data into quartiles. In all statistical analyses (with the exception of bivariate correlations), for normalization purposes, continuous variables were transformed prior to analysis by applying a base 10 (Log10) transformation. *P* < 0.05 was considered significant.

## Results

Of a total available sample of 953 TUDA participants reinvestigated from 2014 to 2018, 875 provided a blood sample, of which 557 participants also provided dietary data. General characteristics of this dietary sub-cohort are shown in Table 2, and those of the original TUDA cohort (sampled 2008 to 2012) and TUDA follow-up cohort (sampled 2014 to 2018) in Supplementary Table 1. Participants were predominantly female (66%) and over 70 y of age (86%). Males, compared with females had significantly higher BMI, waist/hip ratio, and alcohol intake. Dietary intakes of energy and macronutrients were also generally significantly higher in males (Table 2). Potential misreporting of dietary EI was identified as ±30% of the EER value for each individual. Using this approach, a total of 32% of participants reported EIs that were identified as potentially over- or under-reported. As expected [32], most of those misreporting had under-reported EI, and BMI was higher in this group. Notably, however, the data show no significant differences between plausible and misreporters of EI for any of the relevant dietary polyphenol or PVL variables (Supplementary Table 2).

**TABLE 2:** General characteristics and dietary intakes of the TUDA study follow-up sample (n = 557)^1^

	Males (*n* = 187)	Females (*n* = 370)	*P* value^2^
**General characteristics**			
Age, y	76.3 (75.7, 77.0)	75.4 (74.9, 75.9)	0.018
BMI, kg/m^2^	28.9 (28.3, 29.4)	27.4 (26.9, 28.0)	<0.001
Waist/hip ratio, cm	0.98 (0.97, 0.98)	0.89 (0.89, 0.90)	<0.001
Current smoker, *n* (%)	8 (4)	19 (5)	<0.001
Alcohol, units/wk^3^	7.7 (6.0, 9.4)	2.9 (2.5, 3.4)	<0.001
**Dietary intakes**			
Energy (MJ/d)^4^	8.170 (7.872, 8.468)	7.016 (6.842, 7.190)	<0.001
Protein (g/d)	80.8 (78.3, 83.4)	72.0 (70.1, 73.9)	<0.001
Fat (g/d)	73.2 (69.9, 76.5)	65.5 (63.3, 67.6)	<0.001
Saturated fat (g/d)	28.9 (27.4, 30.4)	25.4 (24.4, 26.4)	<0.001
Carbohydrate (g/d)	226.9 (216.7, 237.0)	191.2 (185.9, 196.6)	<0.001
Sugars (g/d)	92.3 (86.9, 97.6)	86.5 (83.0, 89.9)	0.072
Fiber (g/d)	22.0 (21.0, 23.1)	19.4 (18.8, 20.1)	<0.001

**Dietary (poly)phenols**^5^	*mg/d*	***mg/1000kcals/d***	*mg/d*	***mg/1000kcals/d***	
Total (poly)phenols	2336 (2204, 2467)	1281 (1193, 1369)	2256 (2175, 2337)	1416 (1357, 1474)	<0.001
Total flavan-3-ols	608 (556, 650)	336 (306, 365)	708 (674, 741)	435 (413, 457)	<0.001
Total theaflavins + thearubigins	543 (492, 593)	301 (276, 334)	607 (565, 650)	372 (345, 399)	0.114
Theaflavins	72 (65, 79)	40 (36, 44)	81 (75, 86)	49 (46, 53)	0.047
Thearubigins	471 (427, 515)	261 (232, 290)	527 (490, 564)	323 (299, 346)	0.109
Total monomers + proanthocyanidins	536 (499, 573)	295 (270, 321)	627 (598, 656)	386 (366, 405)	<0.001
Monomers	316 (289, 342)	175 (158, 193)	356 (334, 378)	220 (206, 235)	0.002
Proanthocyanidins	220 (198, 243)	120 (107, 133)	271 (253, 289)	165 (154, 177)	<0.001
Total procyanidins + (epi)catechins	146 (136, 156)	80 (73, 87)	155 (147, 162)	95 (90, 101)	<0.001
Procyanidins	86 (80, 92)	48 (43, 52)	90 (85, 94)	55 (52, 58)	0.004
Total (epi)catechins	60 (56, 64)	33 (30, 36)	65 (62, 68)	40 (38, 42)	<0.001
Catechins	23 (21, 25)	13 (12, 14)	24 (23, 26)	15 (14, 16)	<0.001
Epicatechins	37 (34, 39)	20 (19, 22)	41 (39, 43)	25 (24, 26)	<0.001
**Plasma valerolactones (nmol/L)**^6^					
**PVL1:** 5-(Hydroxyphenyl)-γ-VL-sulfate	34.6 (20.7, 48.5)		22.8 (18.9, 26.6)		0.571
*Detected in cohort, n=496 (89%)*					
**PVL2:** 5-(4ʹ-Hydroxyphenyl)-γ-VL-3ʹ-glucuronide	23.5 (17.4, 29.7)		33.5 (24.6, 42.3)		0.069
*Detected in cohort, n=345 (62%)*					
**PVL1+2**	52.0 (35.1, 68.8)		44.2 (36.2, 52.2)		0.286
*Detected in cohort, n=509 (91%)*					
**PVL3:** 5-(5ʹ-Hydroxyphenyl)-γ-VL-3ʹ-glucuronide	8.1 (6.1, 10.1)		10.7 (8.0, 13.3)		0.244
*Detected in cohort, n=178 (32%)*					

1 Data presented are mean (95% CI) unless otherwise indicated. This study involved a new analysis of existing samples from the Trinity-Ulster-Department of Agriculture (TUDA) cohort (*n* = 5186) first sampled from 2008 to 2012 for comprehensive health, but not dietary, data. The TUDA follow-up sample comprises about 20% of the original cohort who were followed up for re-investigation from 2014 to 2018 (*n* = 953); only participants who provided dietary intake data and a corresponding blood sample (*n* = 557) are included in this analysis.

2 Differences in general characteristics between the groups were assessed using independent samples *t* test (continuous variables) or chi-square test (categorical variables). Differences in dietary and valerolactone data were analyzed by ANCOVA, adjusting for age, on log-transformed data where appropriate. *P* value for Dietary (poly)phenols refers to values shown as mg/1000kcals/d. *P* < 0.05 was considered significant.

3 Alcohol consumer; 1 unit equates to 25 mL spirits, 220 mL beer, or 85 mL wine.

4 Potential misreporting of dietary energy intake was identified as ±30% of the estimated energy requirement (EER) value for each individual. Using this approach, a total of 32% of participants reported energy intakes that were identified as potential over- or under-reporters (full details shown in Supplementary Table 2).

5 Dietary (poly)phenol values obtained using a food frequency questionnaire designed specifically to investigate foods containing (poly)phenols, where participants were requested to state the frequency of consumption for food groups or specific products known to contain (poly)phenols.

6 Plasma valerolactone (PVL) metabolites shown here were present in >30% of the samples analyzed. PVL1+2 refers to participants with detectable PVL1 or PVL2 in plasma, but not necessarily both plasma PVLs.

Dietary intakes of (poly)phenols and corresponding plasma PVL concentrations are presented in Table 2. Mean (95% CI) total (poly)phenols intake was estimated as 2283 (2213, 2352) mg/day, of which 674 (648, 701) mg/d were represented as F3O, and 152 (146, 158) mg/d for procyanidins+(epi)catechins. Within this category, monomers and proanthocyanidins were the most consumed, with mean intakes of 343 (325, 360) mg/d and 254 (240, 268) mg/d, respectively. Reported dietary F3O, monomers+proanthocyanidins, and (epi)catechins, intakes were significantly higher in females than in males, whether expressed as mg/d or in nutrient density terms (as mg/1000kcals/d) (Table 2). Regarding plasma PVL concentrations, no significant sex differences were found (Table 2).

Plasma PVL concentrations of the original and follow-up TUDA cohorts (from 5186 and 875 analyzed blood samples, respectively) are presented in Table 3. Of the 9 metabolites targeted, 7 within the current UHPLC-ESI-MS/MS method were identified and quantified in the TUDA participants. Due to the lack of commercially available standards, compound PVL2 was quantified as its isomer, PVL3. Furthermore, it was not possible to distinguish between 5-(3′-hydroxyphenyl)-γ-valerolactone-4′-sulfate and 5-(4′-hydroxyphenyl)-γ-valerolactone-3′-sulfate due to their chromatographic and spectrometric behaviors. Therefore, these 2 last isomers, hereafter called 5-(hydroxyphenyl)-γ-valerolactone-sulfate (PVL1), were quantified by using 5-(3′-hydroxyphenyl)-γ-valerolactone-4′-sulfate as the reference compound (Table 3). Among the identified and quantified metabolites, PVL1 was found in the highest proportion of plasma samples both in the original and follow-up TUDA cohorts (83% and 89%), with mean (95% CI) concentrations of 26.9 (25.4, 28.4) and 26.0 (21.9, 30.2) nmol/L, respectively. In the follow-up investigation, the 3ʹ-glucuronide conjugates of dihydroxyphenyl-γ-valerolactone isomers (PVL2 and PVL3) were present in 62% and 32% of subjects, with mean (95% CI) concentrations of 29.3 (23.9, 34.7) and 9.2 (7.9, 10.4) nmol/L, respectively. All the other metabolites were found in less than 20% of samples from both cohorts (Table 3).

**TABLE 3:** Concentrations of phenyl-γ-valerolactones in plasma samples from the TUDA study^1^

PVL metabolites		Original TUDA cohort (*n* = 5186)			Follow-up cohort (*n* = 875)		
		Detected samples (%)	Mean (95% CI) (nmol/L)	Min-Max (nmol/L)	Detected samples (%)	Mean (95% CI) (nmol/L)	Min-Max (nmol/L)
**PVL1**	5-(Hydroxyphenyl)-γ-valerolactone-sulfate^2^	83	26.9 (25.4, 28.4)	0.8-689.6	89	26.0 (21.9, 30.2)	0.8-938.3
**PVL2**	5-(4ʹ-Hydroxyphenyl)-γ-valerolactone-3ʹ-glucuronide^3^	43	22.7 (21.1, 24.3)	3.0-767.4	62	29.3 (23.9, 34.7)	3.0-1029.9
**PVL3**	5-(5ʹ-Hydroxyphenyl)-γ-valerolactone-3ʹ-glucuronide	13	14.6 (13.1, 16.2)	3.0-158.6	32	9.2 (7.9, 10.4)	3.0-77.5
**PVL4**	5-phenyl-γ-valerolactone-4ʹ-sulfate	5	3.3 (2.9, 3.8)	2.0-28.4	19	3.4 (2.8, 3.9)	2.0-28.1
**PVL5**	5-phenyl-γ-valerolactone-3ʹ-sulfate	2	18.6 (12.8, 24.4)	6.1-321.1	13	7.9 (4.0, 11.8)	2.0-199.9
**PVL6**	5-phenyl-γ-valerolactone-3ʹ-glucuronide	2	37.4 (27.5, 47.3)	15.0-389.1	9	36.8 (28.8, 44.8)	15.0-244.2
**PVL7**	5-(5ʹ-Hydroxyphenyl)-γ-valerolactone-3ʹ-sulfate	<1	62.7 (62.6, 62.7)	62.5-125.0	<1	62.7 (62.5, 63.0)	62.5-125.0
**PVL1+2**^4^		84	38.5 (36.4, 40.6)	2.3-1240.2	90	46.2 (39.7, 52.7)	2.3-1166.4

1 Data presented are mean (95% CI) unless otherwise stated. This study involved a new analysis of existing samples from the Trinity-Ulster-Department of Agriculture (TUDA) cohort (*n* = 5186) first sampled in 2008-2012. The TUDA follow-up sample comprises about 20% of the original cohort who were followed up for re-investigation in 2014-2018 (n = 953); only TUDA follow-up participants who provided a blood sample (n = 875) are included in this analysis. PVL, phenyl-γ-valerolactone. Plasma PVL data for the subsample of participants who provided corresponding dietary data (n = 557) are shown in Table 2.

2 5-(Hydroxyphenyl)-γ-valerolactone-sulfate (PVL1) is the sum of two isomers coeluting, 5-(3′-hydroxyphenyl)-γ-valerolactone-4′-sulfate and 5-(4′-hydroxyphenyl)-γ-valerolactone-3′-sulfate.

3 The compound 5-(4ʹ-hydroxyphenyl)-γ-valerolactone-3ʹ-glucuronide (PVL2) has been quantified as 5-(5ʹ-hydroxyphenyl)-γ-valerolactone-3ʹ-glucuronide (PVL3).

4 PVL1 + PVL2 refers to participants with detectable PVL1 or PVL2 in plasma, but not necessarily both plasma PVLs.

**FIGURE 1:**
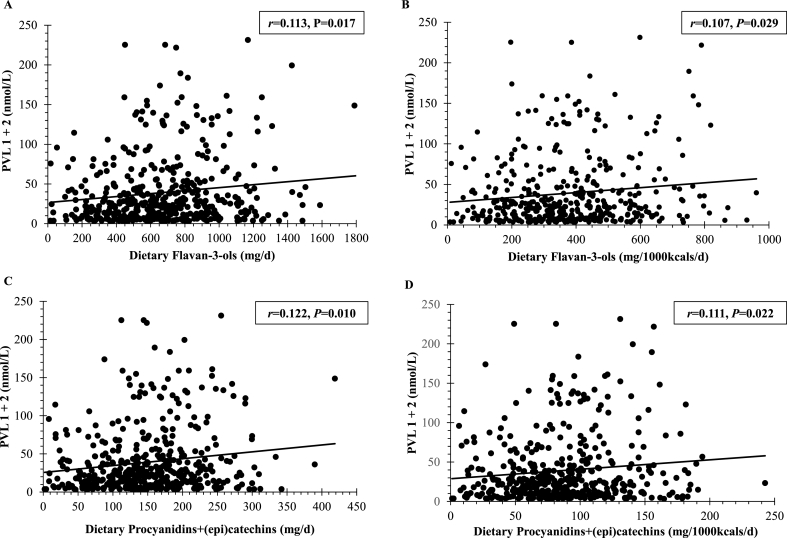
**Association of dietary flavan-3-ols (Upper plots) and procyanidins+(epi)catechins (Lower plots) with plasma phenyl-γ-valerolactone (PVL1+2) concentrations.** Correlations were calculated by using the Spearman correlation coefficient (*r*). Dietary flavan-3-ols and procyanidins+(epi)catechins were each expressed as absolute values (mg/d; panels A and C) and in nutrient density terms (mg/1000kcals/d; panels B and D). PVL1+2 is the sum of PVL1 and PVL2; PVL1 refers to 5-(hydroxyphenyl)-γ-valerolactone-sulfate, PVL2 refers to 5-(4ʹ-hydroxyphenyl)-γ-valerolactone-3ʹ-glucuronide. Results were similar when partial correlations with adjustment for age, sex, and dietary energy intake (kcals/d) were performed on transformed data (Log10) for dietary flavan-3-ols (*r* = 0.111, *r*^*2*^ = 0.012, *P* = 0.020) and procyanidins+(epi)catechins (*r* = 0.119, *r*^*2*^ = 0.014, *P* = 0.012). *P* < 0.05 was considered significant.

**FIGURE 2:**
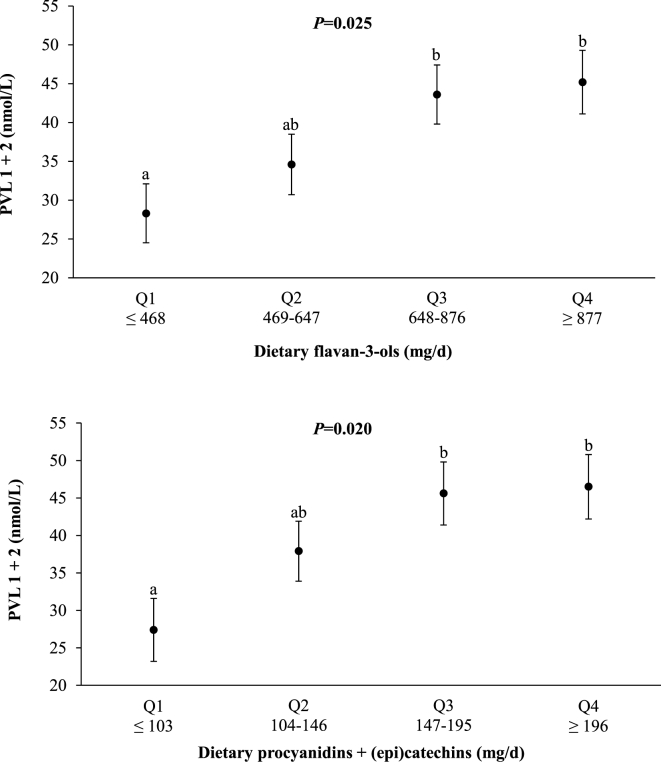
**Plasma phenyl-γ-valerolactone (PVL1+2) concentrations in relation to quartiles of dietary flavan-3-ols (Upper plot) and procyanidins+(epi)catechins (Lower plot).** Values are adjusted mean (SEM) plasma PVL concentrations. PVL1+2 is the sum of PVL1 and PVL2; PVL1 refers to 5-(hydroxyphenyl)-γ-valerolactone-sulfate, PVL2 refers to 5-(4ʹ-hydroxyphenyl)-γ-valerolactone-3ʹ-glucuronide. Dietary data are shown in quartiles ranging from Q1 (lowest 25%) to Q4 (highest 25%) of reported intakes; the range of dietary intakes defining each quartile is shown beneath the corresponding plasma PVL value. The *P* values above each plot refer to differences in plasma PVL concentrations across the quartiles; analysis of covariance (ANCOVA), with adjustment for dietary energy intake (kcals/d). Quartiles without a common superscript letter are significantly different; the Least Significant Difference is the post hoc test. For dietary total F3O, plasma PVL1+2 values (adjusted mean [95%CI]) were 28.3 [20.8, 35.9] nmol/L for Q1 and 45.2 [37.2, 53.2] nmol/L for Q4. For dietary procyanidins+(epi)catechins, plasma PVL1+2 values were 27.4 [19.1, 35.8] nmol/L for Q1 and 46.5 [38.2, 54.9] nmol/L for Q4. Q, quartile. *P* < 0.05 was considered significant.

In correlation analysis using data from participants who provided dietary and corresponding PVL data (n = 557), self-reported dietary intakes of total F3O and procyanidin+(epi)catechin each showed statistically significant correlations with the sum of PVL1 and PVL 2 (PVL1+2) when expressed as absolute values (F3O: *r* = 0.113, *r*^*2*^ = 0.013, *P* = 0.017; procyanidin+(epi)catechin: *r* = 0.122, *r*^*2*^ = 0.015, *P* = 0.010) or in nutrient density terms (F3O: *r* = 0.107, *r*^*2*^ = 0.011, *P* = 0.029; procyanidin+(epi)catechin: *r* = 0.111, *r*^*2*^ = 0.012, *P* = 0.022) (Figure 1). The associations of dietary (poly)phenols with corresponding plasma PVL1+2 concentrations were further examined by grouping participants according to quartiles of dietary F3O and procyanidins+(epi)catechins, ranging from Q1 (lowest 25%) to Q4 (highest 25%) of reported intakes, as depicted in Figure 2. Mean PVL1+2 concentrations increased with increasing quartiles of total dietary F3O, from 28.3 (95% CI: 20.8, 35.9) nmol/L for Q1 (<468 mg/d) to 45.2 (37.2, 53.2) nmol/L for Q4 (>877 mg/d); ANCOVA with adjustment for energy (kcals/d). Likewise, mean plasma PVL1+2 increased with dietary procyanidins+(epi)catechins, from 27.4 (19.1, 35.8) nmol/L in Q1 (<103 mg/d) to 46.5 (38.2, 54.9) nmol/L in Q4 (>196 mg/d).

## Discussion

The current study presents PVL concentrations in over 5000 community-dwelling older adults and follow-up analysis in almost 20%, of which a subsample (n = 557) also provided corresponding dietary F3O-rich food intake data. Our analysis identified 2 specific PVL metabolites, 5-(hydroxyphenyl)-γ-valerolactone-sulfate (PVL1) and 5-(4ʹ-hydroxyphenyl)-γ-valerolactone-3ʹ-glucuronide (PVL2), that were detectable in blood samples from the majority of participants (>62%), whereas the remaining detected PVLs were present in far fewer participant samples (1%–32%) at either time-point. Particularly, we focused on F3O and procyanidines+(epi)catechins due to a better estimation of the intakes of these specific dietary components and, above all, because of the gut microbial metabolism of such compounds where PVLs are mainly produced. We show statistically significant, albeit weak, correlations between reported dietary F3O and procyanidins+(epi)catechins-rich food intakes and plasma concentrations of the sum of the 2 predominant PVLs (PVL1+2).

Mean dietary intakes were estimated as 2283 mg/d for total (poly)phenols, 674 mg/d for total F3O, and 152 mg/d for procyanidins+(epi)catechins, and in the case of F3O, were higher in females than in males (708 vs. 608 mg/d, *P* < 0.001), a sex difference which remained even after dietary energy was considered. Reported dietary (poly)phenol-rich food intakes, including F3O, monomers, oligomers, and polymers, appear to differ widely among European countries. In particular, using population-based data from the UK National Diet and Nutrition Survey (NDNS; n =1724), a median intake value for F3O of 375 mg/d was estimated for British adults [32], whereas F3O intakes were estimated at 650 mg/d for Irish adults using dietary data from the North/South of Ireland Food Consumption Survey (NSIFCS; [33]). In close agreement with the NSIFCS data, the current study of older adults participating in the TUDA study (also incorporating both jurisdictions in Ireland) showed total F3O intakes of 674 mg/d. Most of the dietary F3O intake is represented by theaflavins, thearubigins, and galloyl monomers, compounds for which black tea is the major source in Irish and British diets. As regards total (poly)phenol intakes, the EPIC study estimated these at 1700 mg/d in British adults of 35 to 74 y [34], whereas the current analysis shows considerably higher mean intakes of 2283 mg/d in Irish adults of 65 to 96 y. The explanation for the differences in estimates of (poly)phenol intake between studies likely reflects differences in the dietary assessment methodology used (more comprehensive here compared with the 24-h dietary recall in the case of EPIC; [34]), and/or changes over time (2014-2018 vs. the 1990s). It is also possible that there are real differences in (poly)phenol intakes between these populations, perhaps related to differences in the age profiles, e.g., higher tea consumption in our study participants compared with the younger British adults.

The current analysis of plasma PVLs provides data on over 5000 adults, about 20% of whom were reanalyzed 7 years after initial sampling. We found that the correlations between self-reported dietary F3O and procyanidin+(epi)catechin-rich food intakes and plasma PVL1+2 were statistically significant but weak. Intervention studies have evaluated the metabolic fate of F3O metabolites from different food sources [[35], [36], [37], [38]], but very few previous studies reported values for plasma PVL concentrations at baseline. One small trial (n=10) investigated plasma concentrations of some phenolic metabolites and specific PVLs following the consumption of varying concentrations of total (poly)phenols in cranberry juice [39]. Specifically, volunteers refrained from consuming phenolic-rich foods for 72 h, and their fasting concentrations of 5-(3ʹ-hydroxyphenyl)-γ-valerolactone-4ʹ-sulfate ranged from 0 to ∼150 nmol/L; several of the individual compounds analyzed in plasma were absorbed in a dose-dependent manner [39]. In a crossover study conducted on 12 Dutch males, where intervention (with black tea) was commenced following a 2-day low-(poly)phenol diet, the mean fasting plasma concentration of PVL2 was 2.5 nmol/L [38]. Interindividual variation in circulating metabolites in the latter study was explained by differences in gut microbial PVL production [38]. In good agreement with the current results showing mean values of 26.9 nmol/L and 22.7 nmol/L for PVL1 and PVL2, respectively, in an intervention study where 42 volunteers were fed cocoa with skimmed milk or placebo (skimmed milk only) for 4 weeks, Urpi-Sarda et al. reported a mean fasting plasma concentration of 22 nmol/L for PVL2 in the placebo group [40].

A landmark study by Ottaviani and collaborators [38] examined the absorption, distribution, metabolism, and excretion of epicatechin in 8 volunteers who were provided with 50 mL of a beverage containing 207 μmol of the radioactive isotope [2-^14^C](−)-epicatechin. Urine analysis showed 82% absorption of the [2-^14^C](−)-epicatechin after 48 h ingestion, 42% of which were PVLs (mainly 5-(4′-hydroxyphenyl)-γ-valerolactone-3′-sulfate) and phenyl valeric acids. Plasma and urine analysis were consistent in terms of the types of metabolites identified and, ∼6 h after consumption, the sum of all measured PVLs reached a C_max_ of 588 nmol/L, with 5-(4′-hydroxyphenyl)-γ-valerolactone-3′-sulfate and PVL2 showing the highest concentrations, 272 nmol/L, and 125 nmol/L, respectively [38]. Such values are in good agreement with the concentration range found in the current study.

It has been previously reported that individuals generate different amounts of PVLs following the same intake of F3O owing to differences in the gut microbiota [7,41]. Indeed, Borges et al. recently reported a ∼3-fold difference in total PVLs between human participants following an equimolar intake of *radiolabelled* (−)-epicatechin [7]. Moreover, there are differences in bioavailability of the different F3O stereoisomers and compounds. Ottaviani et al. fed equal quantities of (−)-epicatechin, (+)-epicatechin, (+)-catechin, and (−)-catechin to humans and measured the resulting plasma profiles and reported not only differences in the bioavailability between molecules but also differences in the metabolic fate of the molecules [42]**.** Despite the range of factors that can influence PVL responses to F3O intake, it is worth noting our finding of weak but statistically significant correlations of dietary F3O intakes with plasma PVLs, along with measurable differences in the concentrations of PVLs by quartiles of dietary total F3O or procyanidin+(epi)catechins. The current study, however, cannot validate plasma PVLs as biomarkers of the relevant dietary polyphenols; this can only be addressed in future controlled feeding studies such as that previously reported by Lampe et al. for a range of other potential nutrient biomarkers [43]. The current results provide a first step to enable further investigation of cognitive and other health outcomes linked with the presence and bioactivity of F3O colonic metabolites in the human body, as per our VALID project goals [18]. Future analyses from the VALID project will investigate whether plasma PVLs are associated with cognitive function in older adults while accounting for important socioeconomic and other parameters and thus build on existing in vivo evidence [3,[44], [45], [46], [47], [48]].

The main strength of this study is that it provides data from a comprehensively characterized cohort of over 5000 community-dwelling older adults, with follow-up sampling in almost 20%. This resource provided a unique platform to characterize dietary intakes with regard to F3O (a main class of dietary (poly)phenols) and circulating PVLs, thus contributing considerably to the evidence base in (poly)phenol research. However, this study also has limitations. Most notably, a study of this nature cannot validate plasma PVLs as biomarkers of the relevant dietary polyphenols. Also, the blood samples from TUDA participants, as used in the current analysis, were collected in a nonfasting state, and information on most recent food and drink intakes of (poly) phenol-rich sources prior to sampling was not sufficiently recorded, potentially introducing variability. A major limitation is the well-recognized lack of reliability in reported dietary intake of bioactives using a self-administered FFQ. This, in turn, compromises any analysis and interpretation of the relationship of (poly)phenol intakes with plasma PVLs, considering that PVLs provide an objective measurement, whereas dietary data are inherently problematic and cannot be measured reliably [49]. Given these limitations, particularly the general challenges in accurately assessing dietary (poly)phenols from self-reported records, future controlled human feeding studies are required to validate plasma PVLs as objective measures of dietary F3O intake.

In conclusion, from a panel of 9 potential phenolic metabolites, only 2 specific PVLs were detected in the majority of plasma samples from a large cohort of older adults measured at 2 time points. The sum of these PVLs was found to be weakly associated with reported dietary intakes of total F3O and procyanidins+(epi)catechins. Future studies, in the form of controlled feeding trials, are required to assess the validity of plasma PVLs as biomarkers of these dietary polyphenols.

## Acknowledgments

The authors’ responsibilities were as follows —DDR, HM, AMM, CIRG, and DA conceptualized and designed the study; HM, DDR, AMM, CIRG, DA, PM, MW, JJS, CC, KM, and AJM obtained study funding and provided supervision to researchers at participating institutions; DA and PM performed the laboratory analysis of plasma phenyl-γ-valerolactones; AC, KM, AR, EL, KB, BM, BP, and HJ collected and/or analyzed dietary data under the advice and guidance of LH; AC, MC, and DA performed statistical analyses; DA and AC co-wrote the manuscript; HM, LH, PM, and DDR helped with writing and revising the manuscript for important intellectual content; all authors provided critical feedback and approved the final manuscript; DDR has primary responsibility for the final content.

### Author disclosures

The authors report no conflicts of interest.

### Funding

The VALID Project was awarded under the international Joint Programming Initiative a Healthy Diet for a Healthy Life co-funded ERA-HDHL call on ‘Biomarkers for Nutrition and Health,’ involving partners from the United Kingdom, Italy, and Ireland: Ulster University, Northern Ireland, UK – Biotechnology and Biological Sciences Research Council (Grant BB/P028225/1; Prof. Helene McNulty, overall Project Coordinator); University of Parma, Italy—Ministry of Agricultural, Food and Forestry Policies (Grant DM 31967/7303/16; Prof. Daniele Del Rio, PI); and Trinity College Dublin, Ireland—Science Foundation Ireland (Grant 16/ERA-HDHL/3361; Prof. Anne Molloy, PI).

### Data sharing

Data described in the manuscript, code book, and analytic code will be made available upon request, subject to formal application and approval by the TUDA study consortium.
