# Supplementary material for: Association of dietary flavan-3-ol intakes with plasma phenyl-γ-valerolactones: analysis from the TUDA cohort of healthy older adults
Source: Am J Clin Nutr. 2023 Jun 10;118(2):476–84. doi: 10.1016/j.ajcnut.2023.06.006 (PMC10493433; doi:10.1016/j.ajcnut.2023.06.006)
Supplement: Multimedia component 1 [file mmc1.pptx]

## Slide 1
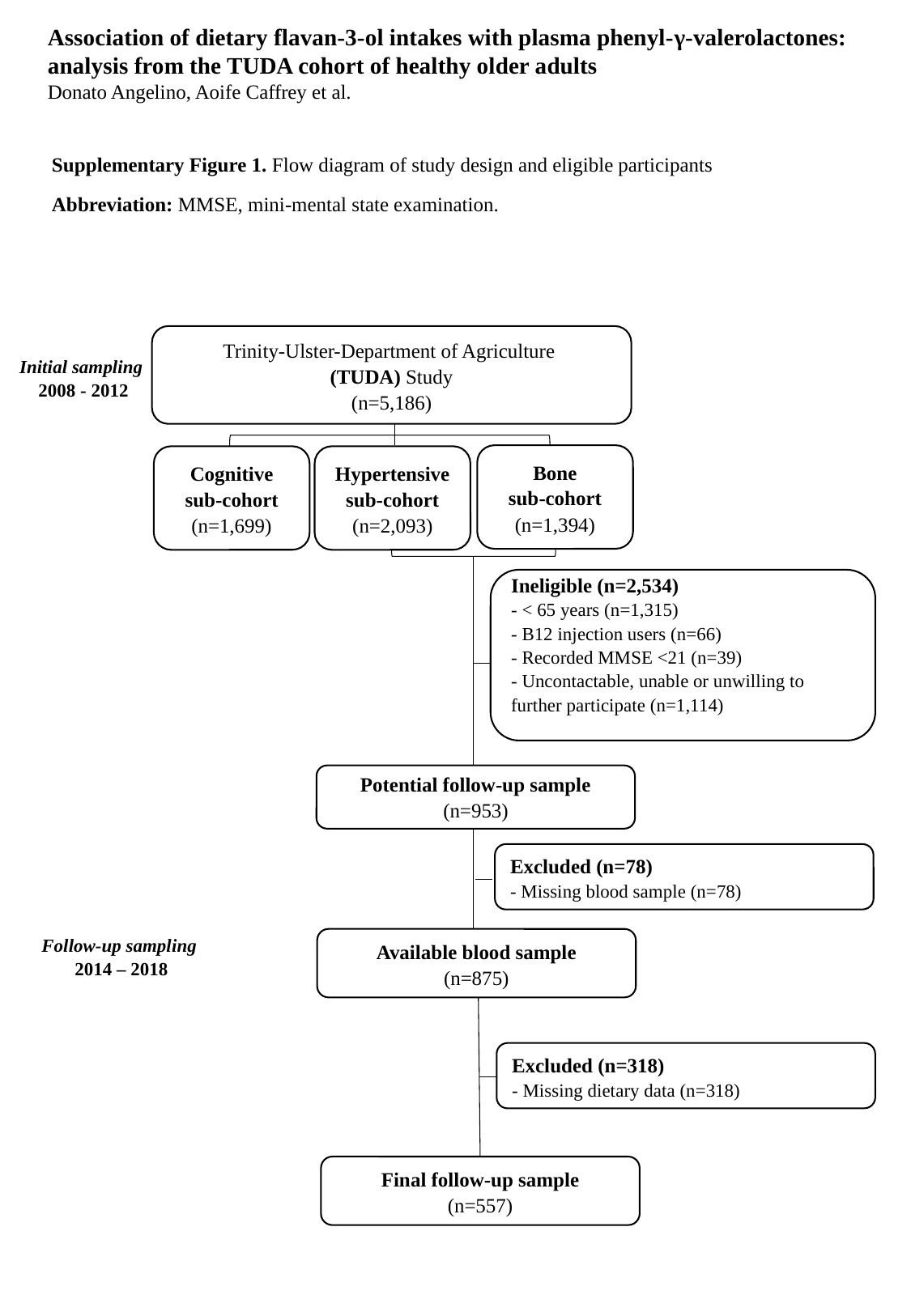

Association of dietary flavan-3-ol intakes with plasma phenyl-γ-valerolactones: analysis from the TUDA cohort of healthy older adults
Donato Angelino, Aoife Caffrey et al.
Supplementary Figure 1. Flow diagram of study design and eligible participants
Abbreviation: MMSE, mini-mental state examination.
Trinity-Ulster-Department of Agriculture
(TUDA) Study
(n=5,186)
Bone
sub-cohort
(n=1,394)
Cognitive
sub-cohort
(n=1,699)
Hypertensive
sub-cohort
(n=2,093)
Ineligible (n=2,534)
- < 65 years (n=1,315)
- B12 injection users (n=66)
- Recorded MMSE <21 (n=39)
- Uncontactable, unable or unwilling to further participate (n=1,114)
Potential follow-up sample
(n=953)
Excluded (n=78)
- Missing blood sample (n=78)
Available blood sample
(n=875)
Final follow-up sample
(n=557)
Initial sampling
2008 - 2012
Follow-up sampling
2014 – 2018
Excluded (n=318)
- Missing dietary data (n=318)

## Slide 2
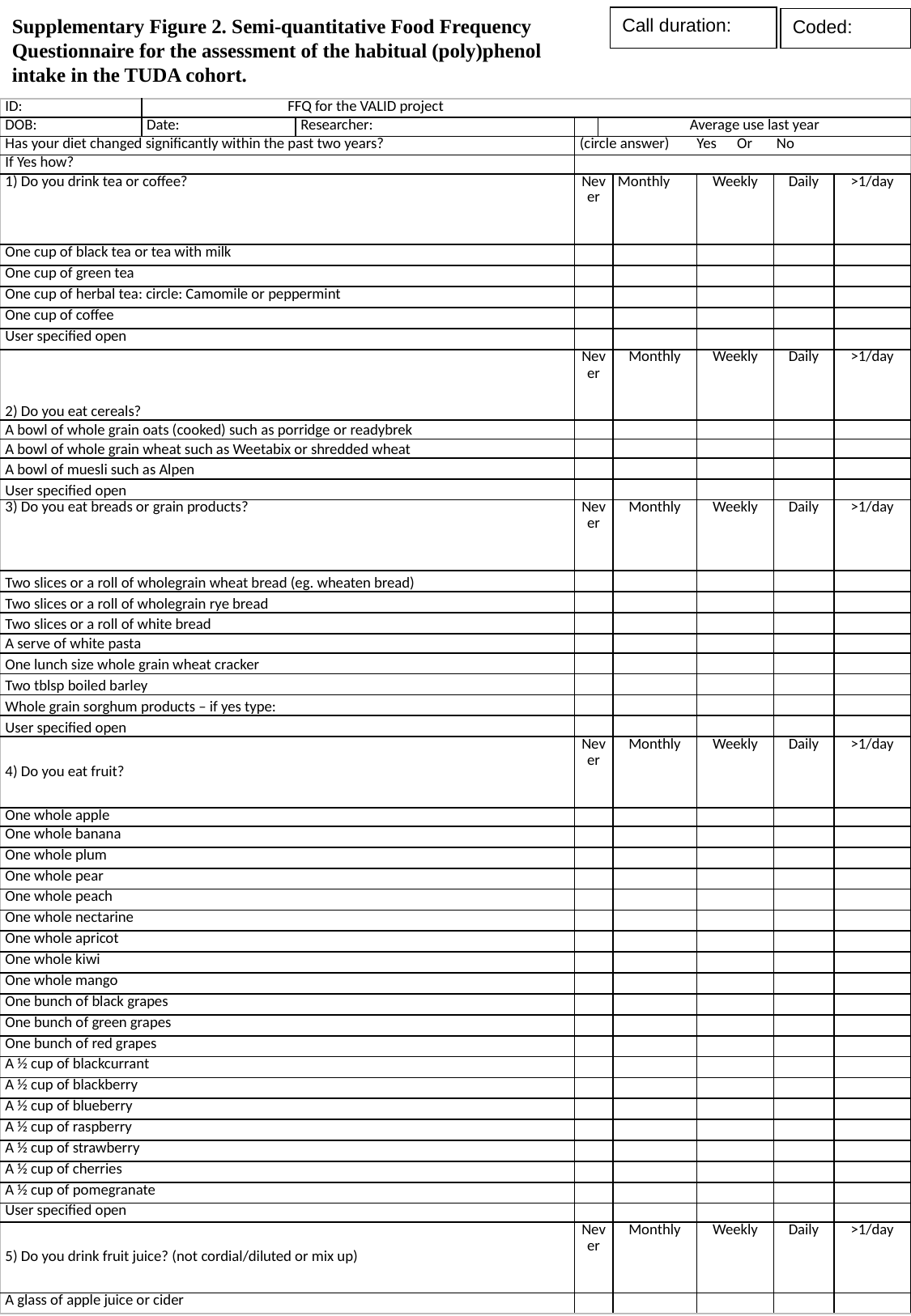

Supplementary Figure 2. Semi-quantitative Food Frequency Questionnaire for the assessment of the habitual (poly)phenol intake in the TUDA cohort.
Call duration:
Coded:
| ID: | FFQ for the VALID project | | | | | | | |
| --- | --- | --- | --- | --- | --- | --- | --- | --- |
| DOB: | Date: | Researcher: | | Average use last year | | | | |
| Has your diet changed significantly within the past two years? | | | (circle answer) Yes Or No | | | | | |
| If Yes how? | | | | | | | | |
| 1) Do you drink tea or coffee? | | | Never | | Monthly | Weekly | Daily | >1/day |
| One cup of black tea or tea with milk | | | | | | | | |
| One cup of green tea | | | | | | | | |
| One cup of herbal tea: circle: Camomile or peppermint | | | | | | | | |
| One cup of coffee | | | | | | | | |
| User specified open | | | | | | | | |
| 2) Do you eat cereals? | | | Never | | Monthly | Weekly | Daily | >1/day |
| A bowl of whole grain oats (cooked) such as porridge or readybrek | | | | | | | | |
| A bowl of whole grain wheat such as Weetabix or shredded wheat | | | | | | | | |
| A bowl of muesli such as Alpen | | | | | | | | |
| User specified open | | | | | | | | |
| 3) Do you eat breads or grain products? | | | Never | | Monthly | Weekly | Daily | >1/day |
| Two slices or a roll of wholegrain wheat bread (eg. wheaten bread) | | | | | | | | |
| Two slices or a roll of wholegrain rye bread | | | | | | | | |
| Two slices or a roll of white bread | | | | | | | | |
| A serve of white pasta | | | | | | | | |
| One lunch size whole grain wheat cracker | | | | | | | | |
| Two tblsp boiled barley | | | | | | | | |
| Whole grain sorghum products – if yes type: | | | | | | | | |
| User specified open | | | | | | | | |
| 4) Do you eat fruit? | | | Never | | Monthly | Weekly | Daily | >1/day |
| One whole apple | | | | | | | | |
| One whole banana | | | | | | | | |
| One whole plum | | | | | | | | |
| One whole pear | | | | | | | | |
| One whole peach | | | | | | | | |
| One whole nectarine | | | | | | | | |
| One whole apricot | | | | | | | | |
| One whole kiwi | | | | | | | | |
| One whole mango | | | | | | | | |
| One bunch of black grapes | | | | | | | | |
| One bunch of green grapes | | | | | | | | |
| One bunch of red grapes | | | | | | | | |
| A ½ cup of blackcurrant | | | | | | | | |
| A ½ cup of blackberry | | | | | | | | |
| A ½ cup of blueberry | | | | | | | | |
| A ½ cup of raspberry | | | | | | | | |
| A ½ cup of strawberry | | | | | | | | |
| A ½ cup of cherries | | | | | | | | |
| A ½ cup of pomegranate | | | | | | | | |
| User specified open | | | | | | | | |
| 5) Do you drink fruit juice? (not cordial/diluted or mix up) | | | Never | | Monthly | Weekly | Daily | >1/day |
| A glass of apple juice or cider | | | | | | | | |

## Slide 3
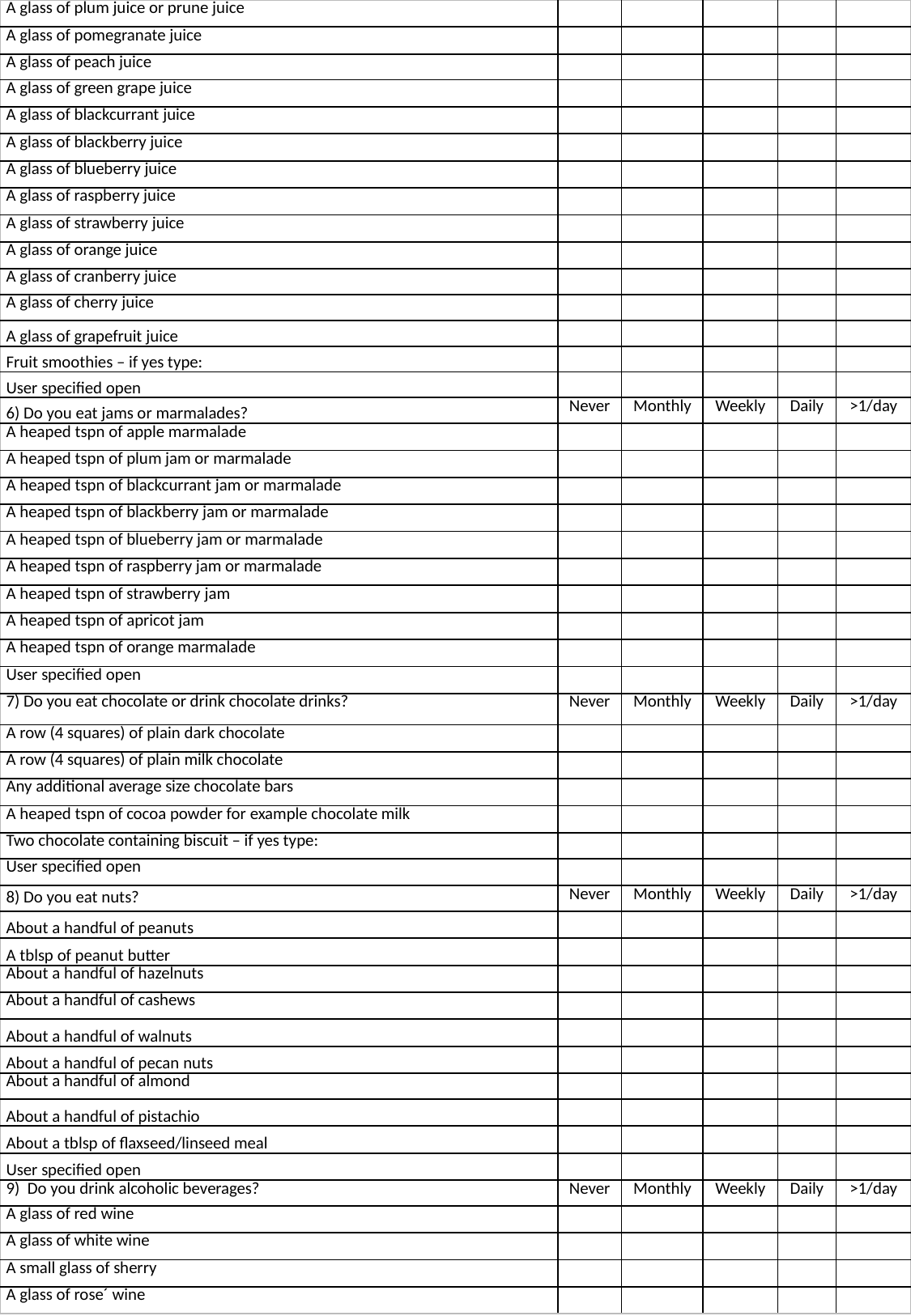

| A glass of plum juice or prune juice | | | | | |
| --- | --- | --- | --- | --- | --- |
| A glass of pomegranate juice | | | | | |
| A glass of peach juice | | | | | |
| A glass of green grape juice | | | | | |
| A glass of blackcurrant juice | | | | | |
| A glass of blackberry juice | | | | | |
| A glass of blueberry juice | | | | | |
| A glass of raspberry juice | | | | | |
| A glass of strawberry juice | | | | | |
| A glass of orange juice | | | | | |
| A glass of cranberry juice | | | | | |
| A glass of cherry juice | | | | | |
| A glass of grapefruit juice | | | | | |
| Fruit smoothies – if yes type: | | | | | |
| User specified open | | | | | |
| 6) Do you eat jams or marmalades? | Never | Monthly | Weekly | Daily | >1/day |
| A heaped tspn of apple marmalade | | | | | |
| A heaped tspn of plum jam or marmalade | | | | | |
| A heaped tspn of blackcurrant jam or marmalade | | | | | |
| A heaped tspn of blackberry jam or marmalade | | | | | |
| A heaped tspn of blueberry jam or marmalade | | | | | |
| A heaped tspn of raspberry jam or marmalade | | | | | |
| A heaped tspn of strawberry jam | | | | | |
| A heaped tspn of apricot jam | | | | | |
| A heaped tspn of orange marmalade | | | | | |
| User specified open | | | | | |
| 7) Do you eat chocolate or drink chocolate drinks? | Never | Monthly | Weekly | Daily | >1/day |
| A row (4 squares) of plain dark chocolate | | | | | |
| A row (4 squares) of plain milk chocolate | | | | | |
| Any additional average size chocolate bars | | | | | |
| A heaped tspn of cocoa powder for example chocolate milk | | | | | |
| Two chocolate containing biscuit – if yes type: | | | | | |
| User specified open | | | | | |
| 8) Do you eat nuts? | Never | Monthly | Weekly | Daily | >1/day |
| About a handful of peanuts | | | | | |
| A tblsp of peanut butter | | | | | |
| About a handful of hazelnuts | | | | | |
| About a handful of cashews | | | | | |
| About a handful of walnuts | | | | | |
| About a handful of pecan nuts | | | | | |
| About a handful of almond | | | | | |
| About a handful of pistachio | | | | | |
| About a tblsp of flaxseed/linseed meal | | | | | |
| User specified open | | | | | |
| 9) Do you drink alcoholic beverages? | Never | Monthly | Weekly | Daily | >1/day |
| A glass of red wine | | | | | |
| A glass of white wine | | | | | |
| A small glass of sherry | | | | | |
| A glass of rose´ wine | | | | | |

## Slide 4
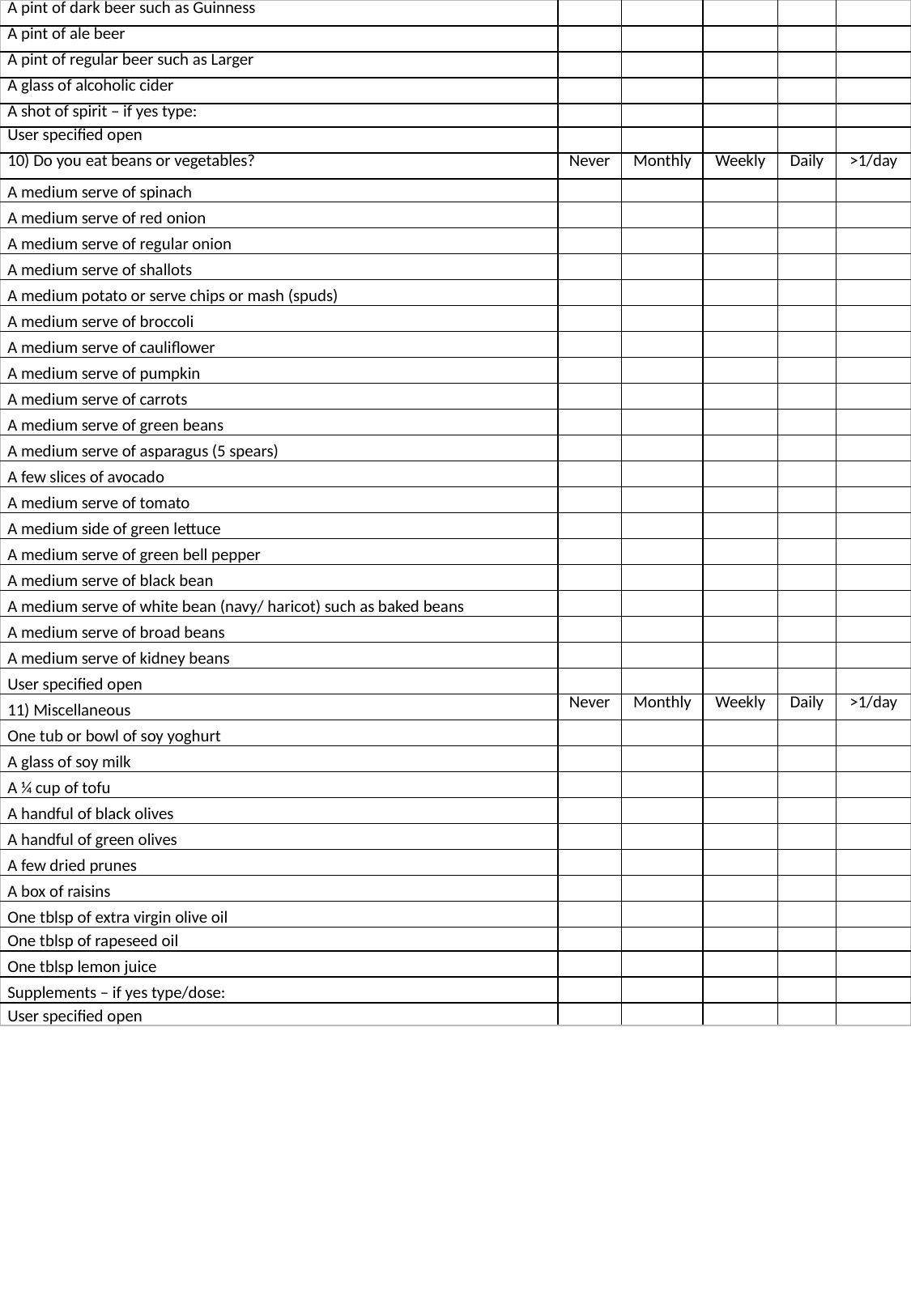

| A pint of dark beer such as Guinness | | | | | |
| --- | --- | --- | --- | --- | --- |
| A pint of ale beer | | | | | |
| A pint of regular beer such as Larger | | | | | |
| A glass of alcoholic cider | | | | | |
| A shot of spirit – if yes type: | | | | | |
| User specified open | | | | | |
| 10) Do you eat beans or vegetables? | Never | Monthly | Weekly | Daily | >1/day |
| A medium serve of spinach | | | | | |
| A medium serve of red onion | | | | | |
| A medium serve of regular onion | | | | | |
| A medium serve of shallots | | | | | |
| A medium potato or serve chips or mash (spuds) | | | | | |
| A medium serve of broccoli | | | | | |
| A medium serve of cauliflower | | | | | |
| A medium serve of pumpkin | | | | | |
| A medium serve of carrots | | | | | |
| A medium serve of green beans | | | | | |
| A medium serve of asparagus (5 spears) | | | | | |
| A few slices of avocado | | | | | |
| A medium serve of tomato | | | | | |
| A medium side of green lettuce | | | | | |
| A medium serve of green bell pepper | | | | | |
| A medium serve of black bean | | | | | |
| A medium serve of white bean (navy/ haricot) such as baked beans | | | | | |
| A medium serve of broad beans | | | | | |
| A medium serve of kidney beans | | | | | |
| User specified open | | | | | |
| 11) Miscellaneous | Never | Monthly | Weekly | Daily | >1/day |
| One tub or bowl of soy yoghurt | | | | | |
| A glass of soy milk | | | | | |
| A ¼ cup of tofu | | | | | |
| A handful of black olives | | | | | |
| A handful of green olives | | | | | |
| A few dried prunes | | | | | |
| A box of raisins | | | | | |
| One tblsp of extra virgin olive oil | | | | | |
| One tblsp of rapeseed oil | | | | | |
| One tblsp lemon juice | | | | | |
| Supplements – if yes type/dose: | | | | | |
| User specified open | | | | | |
