# Supplementary material for: Association of dietary flavan-3-ol intakes with plasma phenyl-γ-valerolactones: analysis from the TUDA cohort of healthy older adults
Source: Am J Clin Nutr. 2023 Jun 10;118(2):476–84. doi: 10.1016/j.ajcnut.2023.06.006 (PMC10493433; doi:10.1016/j.ajcnut.2023.06.006)
Supplement: Multimedia component 2 [file mmc2.docx]

**Association of dietary polyphenols with plasma phenyl-γ-valerolactones: analysis from the TUDA cohort of healthy older adults**

Donato Angelino, Aoife Caffrey et al.

**Supplementary Methods. Chromatographic and mass spectrometry conditions for identification and quantification of phenyl-γ-valerolactone metabolites in human plasma samples.**

Chromatographic and mass spectrometry conditions were set-up in agreement with Brindani et al. 2017. Separations were performed with a Kinetex EVO C18 (100 × 2.1 mm), 2.6 𝜇m particle size (Phenomenex). For UHPLC, mobile phase A was 0.2% formic acid in water and mobile phase B was acetonitrile containing 0.2% formic acid. The gradient started with 5%B, keeping isocratic conditions for 0.5 min, reaching 95%B at 7 min, followed by 1 min at 95% B and then 4 min at the start conditions to re-equilibrate the column. The flow rate was set at 0.4 mL/min, the injection volume was 5 𝜇L, and the column was thermostatted at 40 °C.

The MS worked in negative ionization mode with capillary temperature at 270 °C, while the source was at 300 °C. The sheath gas flow was 60 units, while auxiliary gas pressure was set to 10 units. The source voltage was 3 kV. Ultra high-purity argon gas was used for collision-induced dissociation (CID). Each synthesized compound was directly infused into the ESI source (5 𝜇g/mL at a flow rate of 10 𝜇L/min) in combined mode with a background mode of 70/30 v/v of phase A/phase B at 0.3 mL/min. Characteristic MS conditions (S-lens RF amplitude voltage and collision energy) were optimized for each phenyl-g-valerolactone. The applied method consisted in the selective determination of each target precursor ion by the acquisition of characteristic product ions in the “selected reaction monitoring” (SRM) mode. Two molecular transitions were used to qualify and quantify phenyl-g-valerolactone conjugates. Data processing was performed using Xcalibur^®^ software from Thermo Scientific.

REFERENCE

Brindani N, Mena P, Calani L, Benzie I, Choi S-W, Brighenti F, Zanardi F, Curti C, Del Rio D. Synthetic and analytical strategies for the quantification of phenyl-γ-valerolactone conjugated metabolites in human urine. Mol Nutr Food Res. 2017;61:1700077.

**SUPPLEMENTARY TABLE 1**

General characteristics and dietary intakes of the TUDA study and follow-up cohorts^1^

|  | **All** | **Males** | **Females** | ***P* value^2^** |
| --- | --- | --- | --- | --- |
| **Original TUDA cohort** | (*n*=5186) | (*n*=1699) | (*n*=3487) |  |
| Age, y | 74.0 (73.8, 74.3) | 73.4 (73.1, 73.8) | 74.3 (74.0, 74.6) | <0.001 |
| BMI, kg/m^2^ | 27.9 (27.8, 28.1) | 28.4 (28.3, 28.7) | 27.6 (27.4, 27.8) | <0.001 |
| Waist/hip ratio, cm | 0.91 (0.91, 0.91) | 0.97 (0.97, 0.97) | 0.88 (0.88, 0.88) | <0.001 |
| Current smoker, *n* (%) | 624 (12) | 198 (12) | 426 (12) | <0.001 |
| Alcohol, units/wk^3^ | 7.8 (7.4, 8.3) | 13.7 (12.7, 14.5) | 4.5 (4.2, 4.8) | <0.001 |
| **TUDA follow-up cohort** | (*n*=953) | (*n*=316) | (*n*=637) |  |
| Age, y | 75.8 (75.5, 76.1) | 76.1 (75.6, 76.7) | 75.7 (75.3, 76.1) | 0.089 |
| BMI, kg/m^2^ | 28.1 (27.7, 28.4) | 29.0 (28.5, 29.5) | 27.6 (27.2, 28.0) | <0.001 |
| Waist/hip ratio, cm | 0.93 (0.92, 0.93) | 0.98 (0.97, 0.98) | 0.90 (0.89, 0.91) | <0.001 |
| Current smoker, *n* (%) | 55 (6) | 16 (5) | 39 (6) | <0.001 |
| Alcohol, units/wk^3^ | 4.5 (4.0, 5.0) | 7.8 (6.5, 9.1) | 2.9 (2.5, 3.3) | <0.001 |
| **Dietary sub-cohort** | (*n*=557) | (*n*=187) | (*n*=370) |  |
| Age, y | 75.7 (75.3, 76.1) | 76.3 (75.7, 77.0) | 75.4 (74.9, 75.9) | 0.018 |
| BMI, kg/m^2^ | 27.9 (27.5, 28.3) | 28.9 (28.3, 29.4) | 27.4 (26.9, 28.0) | <0.001 |
| Waist/hip ratio, cm | 0.92 (0.91, 0.93) | 0.98 (0.97, 0.98) | 0.89 (0.89, 0.90) | <0.001 |
| Current smoker, *n* (%) | 27 (5) | 8 (4) | 19 (5) | <0.001 |
| Alcohol, units/wk^3^ | 4.5 (3.9, 5.2) | 7.7 (6.0, 9.4) | 2.9 (2.5, 3.4) | <0.001 |

^1^Data presented are mean (95% CI), unless otherwise indicated. This study involved new analysis of existing samples from the Trinity-Ulster-Department of Agriculture (TUDA) cohort (*n*=5186) first sampled in 2008-2012. The TUDA follow-up cohort comprises about 20% of the original cohort who were followed up for re-investigation in 2014-2018 (*n*=953). The dietary sub-cohort refers to those participants who provided dietary intake data and a corresponding blood sample (*n*=557).

^2^Differences between the groups were assessed using independent samples *t* test (continuous variables), on log transformed data where appropriate, or chi-square test (categorical variables). *P* < 0.05 was considered significant.

^3^Alcohol consumer; 1 unit equates to 25 mL spirits, 220 mL beer, or 85 mL wine.

**SUPPLEMENTARY TABLE 2**

General characteristics and dietary intakes of participants identified as potential mis-reporters compared with plausible reporters of energy intake (n=557)^1^

|  | **All**  (*n*=557) | **Mis-reporters**  (*n*=178) | **Plausible reporters**  (*n*=379) | ***P* value^2^** |
| --- | --- | --- | --- | --- |
| **General characteristics** |  |  |  |  |
| Age, y | 75.7 (75.3, 76.1) | 75.9 (75.2, 76.6) | 75.8 (75.3, 76.3) | 0.353 |
| BMI, kg/m^2^ | 27.9 (27.5, 28.3) | 29.6 (28.9, 30.3) | 27.1 (26.6, 27.6) | <0.001 |
| Waist/hip ratio, cm | 0.92 (0.91, 0.93) | 0.95 (0.93, 0.96) | 0.91 (0.90, 0.92) | <0.001 |
| **Dietary intakes** |  |  |  |  |
| Energy (MJ/d)^3^ | 7.413 (7.253, 7.573) | 6.217 (5.923, 6.512) | 8.015 (7.857) | <0.001 |
| Protein (g/d) | 75.0 (73.5, 76.6) | 66.9 (63.9, 69.8) | 79.2 (77.6, 80.9) | <0.001 |
| Fat (g/d) | 68.1 (66.3, 70.0) | 55.2 (52.0, 58.4) | 74.6 (72.7, 76.5) | <0.001 |
| Carbohydrate (g/d) | 203.5 (198.3, 208.6) | 170.7 (161.6, 179.7) | 220.0 (214.4, 225.6) | <0.001 |
| **Dietary (poly)phenols (mg/d)^4^** | |  |  |  |
| Total (poly)phenols | 2283 (2213, 2352) | 2218 (2097, 2338) | 2338 (2250, 2427) | 0.069 |
| Total flavan-3-ols | 674 (648, 701) | 636 (594, 678) | 693 (658, 727) | 0.274 |
| Total theaflavins + thearubigins | 586 (553, 619) | 564 (512, 615) | 600 (557, 643) | 0.758 |
| Theaflavins | 78 (73, 82) | 75 (68, 82) | 80 (74, 85) | 0.498 |
| Thearubigins | 508 (480, 537) | 489 (444, 534) | 521 (483, 558) | 0.498 |
| Total monomers + proanthocyanidins | 597 (573, 620) | 561 (524, 598) | 613 (583, 643) | 0.245 |
| Monomers | 343 (325, 360) | 328 (301, 355) | 352 (329, 374) | 0.716 |
| Proanthocyanidins | 254 (240, 268) | 233 (210, 256) | 261 (243, 279) | 0.168 |
| Total procyanidins + (epi)catechins | 152 (146, 158) | 145 (135, 155) | 156 (148, 163) | 0.231 |
| Procyanidins | 89 (85, 92) | 85 (79, 91) | 91 (86, 95) | 0.189 |
| Total (epi)catechins | 63 (61, 66) | 60 (56, 64) | 65 (62, 68) | 0.211 |
| Catechins | 24 (23, 25) | 22 (21, 24) | 25 (23, 26) | 0.137 |
| Epicatechins | 39 (38, 41) | 38 (35, 40) | 40 (38, 42) | 0.348 |
| **Plasma valerolactones (nmol/L)^5^** | |  |  |  |
| **PVL1:** 5-(Hydroxyphenyl)-γ-VL-sulfate | 26.9 (21.4, 32.4) | 30.8 (17.7, 43.9) | 25.5 (20.2, 30.7) | 0.865 |
| *Detected in cohort, n=496 (89%)* | |  |  |  |
| **PVL2:** 5-(4ʹ-Hydroxyphenyl)-γ-VL-3ʹ-glucuronide | 29.6 (23.7, 35.6) | 23.9 (17.4, 30.5) | 33.6 (24.7, 42.5) | 0.129 |
| *Detected in cohort, n=345 (62%)* | |  |  |  |
| **PVL1+2** | 46.9 (39.1, 54.8) | 47.7 (31.3, 64.1) | 47.6 (38.6, 56.5) | 0.908 |
| *Detected in cohort, n=509 (91%)* | |  |  |  |
| **PVL3:** 5-(5ʹ-Hydroxyphenyl)-γ-VL-3ʹ-glucuronide | 9.5 (78, 11.3) | 7.1 (5.6, 8.5) | 11.1 (8.4, 13.4) | 0.053 |
| *Detected in cohort, n=178 (32%)* | |  |  |  |

^1^Data presented are mean (95% CI), unless otherwise indicated. This study involved new analysis of existing samples from the Trinity-Ulster-Department of Agriculture (TUDA) cohort (n=5186) first sampled in 2008-2012. The TUDA follow-up sample comprises about 20% of the original cohort who were followed up for re-investigation in 2014-2018 (n=953); only participants who provided dietary intake data and a corresponding blood sample (n=557) are included in this analysis.

^2^Differences in general characteristics between the groups were assessed using independent samples *t* test. Differences in dietary and valerolactone data were analyzed by ANCOVA, adjusting for age and sex, on log transformed data where appropriate. *P* < 0.05 was considered significant.

^3^Potential mis-reporting of dietary energy intake was identified as +/- 30% of EER. A total of 32% of participants reported energy intakes that were identified as potential over- or under-reporters. The data show no significant differences between plausible and mis-reporters of Energy for any of the Polyphenol dietary or biomarker variables.

^4^Dietary (poly)phenol values obtained using a food frequency questionnaire designed specifically to investigate foods containing (poly)phenols, where participants were requested to state the frequency of consumption for food groups or specific products known to contain (poly)phenols.

^5^Plasma valerolactone (PVL) metabolites shown here were present in >30% of the samples analyzed. PVL1+2 refers to participants with detectable PVL1 or PVL2 in plasma, but not necessarily both plasma PVLs.
